# Health services supervision in a protracted crisis: a qualitative study into supportive supervision practices in South Sudan

**DOI:** 10.1186/s12913-022-08637-4

**Published:** 2022-10-14

**Authors:** George William Lutwama, Lodi Joseph Sartison, James Onyango Yugi, Taban Nickson Nehemiah, Zechreya Micheal Gwang, Barbara Akita Kibos, Eelco Jacobs

**Affiliations:** 1 KIT Health, Royal Tropical Institute, Mauritskade 63, Amsterdam, 1090 HA The Netherlands; 2 Montrose International, Africa Office, 31b Bukoto Crescent, Naguru, Kampala, P.O. Box 11161, Uganda; 3 Boma Health Initiative Secretariat, Ministry of Health, Juba, South Sudan; 4 Crown Agents Limited, South Sudan Office, Plot 541, Block 3K, 2nd Class Tong Piny, Juba, South Sudan

**Keywords:** Supportive supervision, Health workers, Health managers, Quality improvement, Protracted crisis, Conflict, South Sudan

## Abstract

**Background:**

The health system in South Sudan faces extreme domestic resource constraints, low capacity, and protracted humanitarian crises. Supportive supervision is believed to improve the quality of health care and service delivery by compensating for flaws in health workforce management. This study aimed to explore the current supervision practices in South Sudan and identify areas for quality improvement.

**Methods:**

The study employed qualitative approaches to collect and analyse data from six purposefully selected counties. Data were collected from 194 participants using semi-structured interviews (43 health managers) and focus group discussions (151 health workers). Thematic content analysis was used to yield an in-depth understanding of the supervision practices in the health sector.

**Results:**

The study found that integrated supportive supervision and monitoring visits were the main approaches used for health services supervision in South Sudan. Supportive supervision focused more on health system administration and less on clinical matters. Although fragmented, supportive supervision was carried out quarterly, while monitoring visits were either conducted monthly or ad hoc. Prioritization for supportive supervision was mainly data driven. Paper-based checklists were the most commonly used supervision tools. Many supervisors had no formal training on supportive supervision and only learned on the job. The health workers received on-site verbal feedback and, most times, on-the-job training sessions through coaching and mentorship. Action plans developed during supervision were inadequately followed up due to insufficient funding. Insecurity, poor road networks, lack of competent health managers, poor coordination, and lack of adequate means of transport were some of the challenges experienced during supervision. The presumed outcomes of supportive supervision were improvements in human resource management, drug management, health data reporting, teamwork, and staff respect for one another.

**Conclusion:**

Supportive supervision remains a daunting task in the South Sudan health sector due to a combination of external and health system factors. Our study findings suggest that strengthening the processes and providing inputs for supervision should be prioritized if quality improvement is to be attained. This necessitates stronger stewardship from the Ministry of Health, integration of different supervision practices, investment in the capacity of the health workforce, and health infrastructure development.

**Supplementary Information:**

The online version contains supplementary material available at 10.1186/s12913-022-08637-4.

## Background

South Sudan is ravaged by civil war, natural disasters, extreme health risks, food insecurity, the COVID-19 pandemic, and severe underfunding for basic services [1, 2]. In turn, these challenges have a major impact on health care delivery. Health service delivery has been directly affected by a shortage of health personnel, facilities and resources and a continuous disruption by conflicts [2, 3]. The protracted violent conflict in the country has destroyed livelihoods, caused famine, deteriorated public health and led to the destruction and looting of health facilities, putting further pressure on the already fragile health system [4–6].

Supportive supervision is a process of guiding, helping, training, and encouraging workers to continuously improve their performance in a respectful and nonauthoritarian way [7, 8]. Supportive supervision is vital in the management of health workers and is believed to improve the quality of care and health service delivery [9–11], yet it is challenging to implement due to the low levels of medical training in the country [2, 12]. Supportive supervision is expected to improve the quality of care, which compensates for flaws in health workforce management and training [13–16]. Unlike the traditional supervision approaches of top-down inspection and control, supportive supervision promotes quality improvement by strengthening relationships, communication, mentorship, joint problem-solving and maximizing resource allocation to continuously improve performance [8, 13, 14, 17–19]. Supportive supervision involves three main interrelated functions, namely, administrative, educational, and supportive [8, 13, 20]. The administrative element concerns matters related to policy implementation, the educational component addresses learning and professional development, and the supportive component relate to job stresses, motivation and creating a conducive work environment [13, 15, 16, 18].

Embedding supportive supervision within a quality improvement process fosters accountability, mentorship, trust, teamwork, and open two-way communication. This may increase health workers’ knowledge, skills, confidence, motivation, and focus on performance [8]. When the quality of supervision is poor, the number of supervision visits will have no impact on performance [15, 16]. Effective supportive supervision should focus on specifying the objectives and expectations, monitoring performance, providing motivation, improving job satisfaction, providing targeted training, linking the levels of the health system, replicating best practices, and supporting planning and problem-solving processes [16, 17]. Thus, implementing supportive supervision requires motivated supervisors and health workforce, time investment, authority to make decisions, and tools appropriate for the context [14].

### South Sudan health sector and supervision of health services

As a result of extreme domestic resource constraints, low capacity and protracted crisis, non-governmental (NGOs) and faith-based organisations provide nearly 80% of the health services in South Sudan [4, 6, 21]. The health system is based on three tiers. The first tier is primary care, which includes a community structure known as the Boma^1^ Health Teams, Primary Health Care Units (PHCUs), and Primary Health Care Centres (PHCCs). The second tier is secondary care, which includes county hospitals and state hospitals, and the third tier is tertiary care comprising national teaching, specialist, and referral hospitals. Since 2012, pooled donor funding mechanisms such as the Health Pooled Fund (HPF)^2^ and the World Bank have financed the implementation of the Basic Package of Health and Nutrition Services through contracted NGOs [4, 6, 22]. To strengthen the health systems and improve the quality of care, the Ministry of Health (MoH) introduced the quantified supervisory checklist (QSC) in 2011 [21]. The purpose of the QSC was to systematically identify the successes and the barriers to health service delivery through a joint problem-solving process with the health workers, the County Health Departments (CHD) and the supporting NGOs. The QSC measures seven components, which include equipment, infrastructure, human resources and management, health information management systems, pharmaceuticals, service provision, and utilization of services [23].

The pooled donor funds support the MoH through the contracted NGOs and the CHDs to ensure that supportive supervision is carried out in all health facilities. To that end, since 2015, the HPF has supported the MoH in developing a supportive supervision manual, the Health Sector Quality Improvement Framework [24] and the quality of care (QoC) strategy 2019–2023 [25]. These supervision frameworks were meant to align supportive supervision within the overall health sector reforms to strengthen quality improvement. Despite these efforts, the quality of care is still poor in most health facilities [2, 3, 26–28]. Little is documented about the link between the quality of care and supervision in South Sudan. Although some studies have been conducted concerning supervision of community health workers [12], to our knowledge, supervision at the health facility and the CHD levels in South Sudan have so far remained unexplored in the scholarly literature. It is, therefore, necessary to fill the knowledge gaps and provide insights to policymakers, health programmes and development partners to improve supportive supervision practices and contribute to a more resilient health system. This study aimed at exploring the current supervision practices in South Sudan and identify aspects of quality improvement.

## Methods

### Study design

The study used a qualitative research design, using semi-structured interviews (SSI) and focus group discussions (FGDs) to obtain an in-depth understanding of supervision practices from the different stakeholders in the health sector. This study was guided by three research questions (a) What approaches are used to supervise health services in the counties? (b) What are the experiences of health workers with the current supervision practices? (c) What challenges are faced during supervision and how are they mitigated?

### Study setting

The study was conducted in four states of South Sudan. Three states were selected randomly, and one state was selected purposively. The randomly selected states were Eastern Equatoria, Western Bahr El Ghazal, and Unity. The Central Equatoria state was selected purposefully because it hosts the capital city Juba, the headquarters of the MoH, the donors, and the implementing NGOs. Two counties were purposefully selected for each state using the following criteria: (a) the distance from the state capital - the far and near counties, (b) performance of the county in terms of health data reporting - one good and another poor, and (c) where potential participants were accessible without compromising their and the researchers’ security. The counties selected include Juba and Yei in Central Equatoria, Torit and Kapoeta South in Eastern Equatoria, Rubkona and Pariang in Unity State and Wau and Jur River in Western Bahr El Ghazal State.

### Participants

The study involved 194 participants. Approximately 63% of the participants were men, and 37% were women (Table 1). The 43 SSI participants were health managers from MoH (national, state, county, and hospitals), NGOs, and United Nations (UN) agencies. Thirty-eight FGD participants were professional health workers (e.g., doctors, nurses, clinical officers, midwives, laboratory staff, etc.) from the five hospitals, and 113 were from 14 primary care facilities spread across the four states.

**Table 1:** Distribution of study participants by state, sex, and method of data collection

State	Semi-structured interview participants		Focus group discussion participants		Total Participants
	Male	Female	Male	Female	
Central Equatoria	11	2	25	37	**75**
Eastern Equatoria	7	0	14	6	**27**
Unity	11	1	22	9	**43**
Western Bahr El Ghazal	11	0	22	16	**49**
**Total number of participants**	**40**	**3**	**83**	**68**	**194**

### Selection of the participants

Table 2 summarises the recruitment strategy of the study participants. The health managers were purposefully selected from their respective states, counties, and hospitals under the guidance of the State Ministry of Health (SMoH) and the county health departments (CHD) officials and based on their roles and experiences with the supervision of health services. The health managers were heads of the department from MoH (National, state, county, and hospitals) and non-government health actors, such as UN agencies and NGOs, who are involved in decision making and supervision of the health services. The health workers for the FGDs were also selected purposefully from all departments within the sampled health facilities. The CHD medical officers guided the selection of the health facilities where the FGDs were conducted, while the health facility in-charge guided the researchers in the recruitment of the participants (health workers).

**Table 2:** Recruitment strategy of the respondent groups

Respondent group	Eligibility criteria	Recruitment strategy	Number of participants	The method used for the interview
1. Ministry of Health Officials (National MoH, state MoH, County health department officials, state/county hospital directors/administrators)	Heads of the department and/or their representatives who are involved in the supervision of health services and decision-making.	Purposive selection with support from the National and State Ministry of Health Officials.	21	Semi-structured interviews
2. Non-governmental actors (NGOs, United Nations Agencies, Fund Managers for the pooled funds for the health programmes)	Senior officials from the organisations involved in the provision and supervision of health services in the states/counties. One of the NGOs from each county or state must be an HPF programme implementing partner. The UN agencies must be involved in supporting the health sector.	Purposive selection in consultation with State Ministry of Health Officials, UN Agencies and Fund managers for the pooled funds. The non-HPF implementing NGOs were selected using snowballing sampling.	22	Semi-structured Interviews
3. Hospital health workers (state & county) and PHC health workers from the Primary Health Care Centres (PHCCs)	Male and female health workers - managers/ward in charge, doctors, clinical officers, nurses, midwives, laboratory staff, and other allied health professionals working in the selected hospitals and primary care facilities for at least one year. Stratifying criteria were gender, profession, and position of authority.	Purposive selection in consultation with the health facility in-charge, hospital administrators, and supported by the CHD medical officer.	151	Focus group discussions

The table summaries the processes that were followed during the recruitment of the study participants

### Conceptual framework

**Fig. 1:**
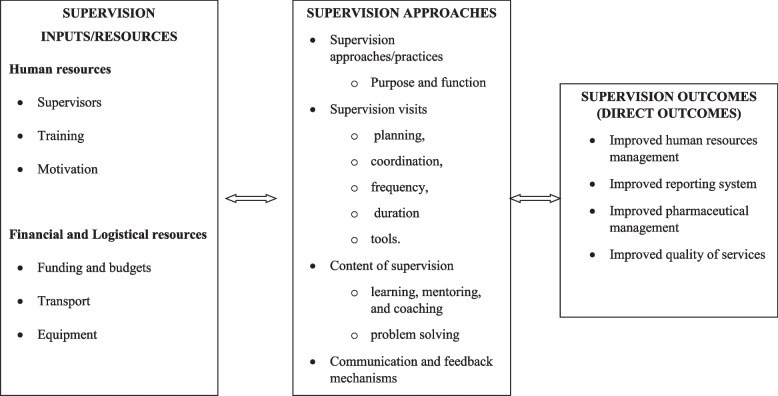
Conceptual framework for supportive supervision of health services in South Sudan. The framework presents the themes and subthemes that guided data collection, analysis, and presentation of the study findings

Supportive supervision is known to enhance professional development, promote personal growth through appraisal, and positively change the work environment [18, 19] Nancarrow et al. [29] identified 13 key domains of a supervision framework. The domains include a clear definition of the type of supervision and support, purpose and function, models (managerial, educational, restorative/supportive), context (where supervision takes place), and the content of supervision. Other domains are modes of engagement during supervision, supervisor attributes, supervisors’ responsibility, supervisory relationships, structures/processes for supervision and support, facilitators and barriers to supervision and support, and the outcomes of supervision. The relationships between supervisors and their subordinates are key to effective supervision. Drawing from this and inspiration from the literature [10, 30], a conceptual framework was developed (Fig. 1) to guide the data collection, analysis, and reporting.

The frameworks consist of three major themes: supervision inputs, process, and outcomes. Each of these themes has several sub-themes. The framework assumes that if effective supportive supervision is to be achieved, there should be inputs to support the processes. The lack of these will prevent supportive supervision from achieving the intended objectives.

### Data collection

The data was collected from 43 SSIs and 19 FGDs between November 5th and December 16th, 2020. FGDs in Kapoeta South County were not conducted due to insecurity and flooded airstrips, which led to the cancellation of air flights. A saturation point was reached by the 38th SSI and the 17th FGD. On average, the interviews took 47 minutes, ranging from 33 to 75 minutes. The average time for the FGDs was 75 minutes, ranging from 57 to 103 minutes. All except one health manager were interviewed face-to-face from their respective workplaces and were moderated by LJS, JOY, TNN, and BAK. GWL provided overall supervision of the fieldwork. One health manager for Kapoeta South County was interviewed using Skype. The moderators were experienced researchers with master’s level education. One researcher was a woman, and the rest were men. Before the fieldwork, the researchers attended a two-day refresher training in Juba facilitated by the first author. The training covered the research background, the research tools, sampling strategy, research ethics and how to conduct interviews and FGDs. The SSI and FGD topic guides were pretested in Juba to understand how they elicited responses to the study objectives.

Semi-structured topic guide (Supplementary file 1) was used to interview the health managers. The topics covered include methods used to conduct supervision (how supervision is conducted, who supervises, frequency of supervision, and training of supervisors), communication and feedback, the challenges, and suggestions for improvement. The researchers followed the guide but were also able to interrogate topical trajectories in the conversation that may stray from the guide when it seemed appropriate.

The FGD topic guide (Supplementary file 2) contained open-ended questions to explore health workers’ experiences with supervision. The FGDs were held in a well-ventilated space/open-air space within the compound of the health facility where privacy was assured. Due to COVID-19 restrictions on gatherings, the number of participants was limited to between six and 10 per group. Physical distancing was ensured, and the use of face masks was encouraged. Hand washing and/or hand sanitising were also encouraged before entering the meeting venue.

All interviews (except two - one each from Wau and Yei counties) and FGDs (except one from Wau county) were audio-recorded with permission from the participants. The audio recordings were supplemented by field notes. All interviews and 14 FGDs from Yei, Torit, Unity State, and Juba were conducted in English. Five FGDs from Pariang, Juba, and Wau counties were conducted in a mixture of Arabic and English. The researchers deployed in these areas were fluent in both English and Arabic; hence, there was no difficulty in moderating and understanding the discussions.

### Data analysis

Verbatim transcription of the recordings was performed in English by an independent consulting firm. The five FGDs that were conducted in a mixture of Arabic and English were also transcribed verbatim in English by one of the co-investigators (ZMG) fluent in both languages and were reviewed by BAK. For the three non-audio recorded interviews, comprehensive notes were taken. The first (GWL) and second authors (LJS) checked all the transcripts for accuracy and completeness. All the transcripts and field notes were imported into NVivo 12 software (QSR International) [31] for coding and analysis. Coding was performed deductively based on the conceptual framework in Fig. 1. Two researchers (JOY and BAK) independently reviewed the codes and the subcategories. Thematic content analysis was performed by the first (GWL) and second authors (LJS). Content analysis aided in identifying the common patterns and trends arising from the data regarding practice and the experiences with supportive supervision in the counties. Narratives were written on the main themes and subthemes, and some quotes were used for illustration in the results section. FGD and SSI data were triangulated to enhance the quality and reliability of the findings.

## Results

The results are presented based on the three major themes in the conceptual framework, which include supervision inputs/resources, supervision approaches, and supportive supervision outcomes.

## Supportive supervision resources/inputs

### Human resources

#### Supportive supervision teams (supervisors)

A multidisciplinary team of professionals from the SMoH, the CHD, UN agencies and the implementing NGOs conduct joint supportive supervision sessions. Each team member is allocated thematic areas to supervise based on her/his expertise (e.g., family planning, child health, nutrition, health data reporting, facility administration, and infection prevention and control). The monitoring visits, on the other hand, are mostly conducted by the CHD, implementing NGOs, fund managers, donors and third-party monitors. Most health workers were satisfied with the supervisors and the support they received. Nearly all the health workers described the supervisors as being polite, friendly, and acting as teachers and not like the police. In some counties, a few supervisors were reported to be harsh. For example, in one of the counties, some health workers said *“… for a small mistake they (supervisors) shout at you or send you packing”* (FGDs #8 & #9). However, some managers mentioned that some counties lack adequate and competent CHD staff to conduct supportive supervision activities. The managers also reported a high turnover of staff at CHD and SMoH, which compromised the consistency in knowledge related to supportive supervision.

#### Training on supportive supervision

The health managers acknowledged receiving either formal or on-the-job training sessions on the supervision of health services. The training sessions on supportive supervision were mostly performed as a component of other training sessions, such as Health Management Information System, leadership and management, and the expanded programme of Immunization (EPI). A few managers received standalone training sessions on the supervision of health services ranging from five to 10 days through MoH collaborative arrangements with the Centre for Disease Control, World Health Organization (WHO), African Field Epidemiology Network, African Medical and Research Foundation, HPF, and United Nations Development Programme. Nearly all the CHD and other health managers interviewed had training on the use of the QoC mobile application and on-the-job training on how to use the quantified supervisory checklists (QSC).

#### Supervisor and supervisee motivation

Many health workers from the FGDs were satisfied with the way supervision was conducted but requested the duration of the visits to be increased so that there is adequate time to discuss, share and jointly develop action plans. There were some health workers from Juba, Torit, Pariang, and Rubkona counties who were not satisfied with supportive supervision because they did not receive feedback. For others, the action plans they developed were not implemented. The managers indicated that some health partners, such as UNICEF and WHO, provide monetary allowances to the supervisors, for example, MoH officials who go for supportive supervision and spend a night out of their duty stations. Where no overnight stay was anticipated, many organisations did not provide any allowances except for refreshments such as water, biscuits and sometimes lunch. Some managers, however, referred to instances where some supervisors refused to participate in supportive supervision activities where no allowances were provided for.

### Financial and logistical resources

#### Funding and implementation of action plans

Generally, supervision is challenged by a lack of funds to execute some action plans, especially those that require money. Most of the supportive supervision initiatives are donor-funded and channelled through the implementing NGOs. There is limited funding from the government to support the supervision of the health service. The managers mentioned that despite having supervision plans, there are inadequate funds to implement the action plans and the recommendations arising from the supportive supervision activities. Such challenges are beyond the control of the CHD and the implementing NGOs, particularly when the recommended activities are outside the donor approved plans and priorities.

#### Transport

There is a lack of reliable means of transport in most counties to facilitate supportive supervision activities. Motor vehicles are the most commonly used means of transport. Most vehicles are very old, and they keep breaking down. These vehicles and fuels are provided by health partners, the implementing NGOs, fund managers’ state teams, UN agencies, the CHD and SMoH. Where there are rivers, in Unity and Central Equatoria states, supervisors use boats (canoes) to cross the rivers. In Torit County, some managers use motorbikes to access health facilities during the rainy season. Due to insecurity in Yei County, motorcycles are only used to supervise facilities within the town. Air transport is used to travel from Juba to the states and/or interstate movement due to insecurity and bad roads. For example, a manager from Torit County said that *“…these days the supervision teams from Torit to Kapoeta travel by air because of the insecurity on the roads and once they reach there, they use a two vehicles convoy movement for security reasons”* (SSI #7).

#### Tools and equipment

Most managers complained of a lack of stationeries for printing the checklists, smartphones, and/or tablets for Open Data Kit (ODK) and QoC mobile applications. Many health facilities also mentioned the lack of essential diagnostic equipment to facilitate service delivery and quality of health care.

### Supervision approaches

Two main supervision approaches were used, namely, supportive supervision and monitoring visits. These included one-to-one and group supervision sessions.

#### Purpose and function

The health managers stated that the current supportive supervision activities focus on the functionality of the whole health system rather than individual health workers and provide some training opportunities. The emphasis is more on the management tasks and less specific on clinical aspects. The supportive supervision visits cover most aspects related to primary care delivery, pharmaceutical management, availability and use of medical equipment, quality of care, and community health initiatives. The monitoring visits are spot checks to verify staff attendance, the functioning of the health facilities, data quality, availability of drugs, and follow-up on action plans from the previous supervision visits as well as to prioritize the locations in need of supportive supervision. From the interviews, it emerged that some health managers could not differentiate whether they were conducting monitoring or supportive supervision visits.

#### Planning and coordination of supervision

Many health workers mentioned that the CHD officials inform them about the planned supportive supervision visits through letters, e-mails or telephone calls. A few health workers from Torit and Yei Counties, however, indicated that some supervisors just show up at the health facilities without notice. The managers mentioned that supportive supervision visits are planned based on the data received from the facilities. Often, supervisory visits also happen because some health facilities have not been visited for a long period. At times, the facilities are visited at the request of the national MoH and UN agencies due to problems reported to them from the community. One manager from Juba County said, *“We visit a facility or a county based on the health data we receive and sometimes due to emergencies”* (SSI #14). In some counties, the locations or health facilities to visit for supportive supervision are selected randomly.

Assembling a team of supervisors for the planned supervision visits was mentioned as a challenge by the health managers. The absence of supervisors when they are required leads to distortion and cancellation of supportive supervision visits. At times, the supervisors either find health facilities closed or staff absent from duty. There was insufficient coordination of supervision activities between the national and sub-national levels. The health workers from the FGDs mentioned that supportive supervision teams visit the facilities in the morning hours when the client load is high, which inconveniences the patients since all the health workers are taken up by the supervisors. At the same time, this limits the duration the supervisors engage with the health workers. As a health worker from Yei County said, *“… at times the supervisors ask us (staff) to release patients to give them (supervisors) time to answer their questions”* (FGD #9). Other health workers from Torit County indicated that *“… sometimes the supervisors come at very late hours for example approximately 3 or 4 pm and hardly spend enough time to listen to our (health workers) challenges”* (FGD #13). The health workers from most FGDs suggested that supervisors should come after midday or a time when the client load is low to have meaningful engagements.

#### Frequency of supportive supervision

Usually, supportive supervision visits take place quarterly, while monitoring visits are done monthly but sometimes ad hoc. It was, however, noted that some facilities are supervised regularly (some take up to 6 months or more to be supervised). Unlike the monthly or ad hoc spot checks, the quarterly supportive supervision visits include on-the-job training sessions on several topics, such as completing registers, the use of clinical guidelines, and infection prevention and control practices.

#### Duration of supportive supervisory visits

The time spent during supportive supervisory visits varies by the type and size of the health facility. The health managers and some health workers indicated that the supervisors spend approximately 30 minutes in PHCUs, two to 4 h in PHCCs and up to 5 h in hospitals. A health manager from Juba County said *“… I (supervisor) have an experience where a PHCU is a small ‘tukul’ (hut) with just one room and maybe with two staff, so that would not take more than an hour to supervise”* (SSI #10). It was not uncommon to find that the supervision teams visited more than two or more facilities per day, especially for the smaller health units.

#### Tools used for supportive supervision

There are multiple tools used by supervisors in health facilities, some of which have similar information. Paper-based checklists are the most commonly used tools during supervisory visits. These checklists include the quantified supervisory checklist (QSC) used quarterly by the MoH, CHD and the implementing NGOs and other programme-specific checklists such as for nutrition used during the monitoring visits. There are also web-based tools, such as the HPF Quality of Care (QoC) mobile application and Open Data Kit (ODK), used to supervise immunization services. Nearly all the health managers indicated that the QSC is user-friendly and less time-consuming. An NGO manager from Yei County said that *“... although there is some information missing in the QSC, we (NGO) have added accessory tools (such as for laboratory, nutrition, de-worming services) to collect relevant data for quality improvement”* (SSI #36). Some health workers also mentioned that some supervisors visit facilities without clear supervision objectives and checklists.

#### Content of supportive supervision

According to the health workers, the supervisors move in teams and visit all departments in the health facilities to look at the registers, outpatient services, maternal and child health services, infection prevention and control, the pharmacy, community health activities, laboratory activities and the general management of the facility. The supervisors also discuss with the health workers the action points arising from the visits through a feedback mechanism. A health worker from Jur River County said that *“… if there is anything to be corrected, the supervisors give immediate on-site guidance to the staff to improve”* (FGD #3).

Nearly all FGD participants acknowledged receiving mentorship and coaching during supportive supervision. For example, mentoring on how to conduct health education sessions, record keeping, including completing the patient registers, updating drug stock cards and de-junking expired drugs. Additionally, during supportive supervision, health workers have opportunities to interact with SMoH, CHD staff and other health partners, such as UN and NGO staff. An FGD participant from a PHCC in Torit County said *“… they (health workers) see the supervision as the climbing a ladder for them (health workers) to correct mistakes and learn”* (FGD #5).

#### Communication and feedback

The health managers mentioned that they mostly gave immediate verbal feedback about their supportive supervision findings. This was corroborated by many health workers during the FGDs. The feedback sessions involve all health facility staff to brief them on the findings and agree on the immediate actions as well as the recommendations for improvement. There were, however, some health workers from Juba, Torit and Rubkona counties who said that they had never received any feedback after supportive supervision. In addition to the verbal feedback, many facilities had supervision registers where supervisors write their findings, recommendations, and action points. The written feedback acts to reinforce the information shared during supportive supervision.

In Rubkona and Yei Counties, the managers also share feedback on the findings from supportive supervision during the county quarterly review meetings. The review meetings are attended by the SMoH and CHD officials, health facility in-charges, fund managers (such as HPF) representatives, NGOs, UN representatives, community and local government leaders. Another avenue for sharing the supervision findings with other health partners and donors is through the health cluster meetings held at the state and national levels.

The managers indicated that the feedback given during and after supportive supervision is well received by most of the health workers since it gives them opportunities to learn new skills and improve their knowledge. A manager from Jur River County said that *“… I have not seen any instances where there is a negative response or any push back from the health workers, because we (supervisors) are part of the same system and figuring out how to improve services delivery”* (SSI #29). However, another manager from Rubkona County stated that *“… there are few health workers who perceive feedback negatively, but it depends on how the supervisors communicate with them”* (SSI #22). Some health workers appreciate their supervisors depending on the way you approach them, the way you coach them, the way you talk to them, and the way you give them feedback. A manager from Juba County said, *“… once you are harsh to the health workers during the feedback session they will fight back”* (SSI #3). In Jur River and Wau counties where written feedback is given, the CHD officials take the lead in sharing the supervision report with the health facilities and the SMoH. At the facility level, the in-charge is responsible for communicating the supervision findings to her/his staff.

#### Problem-solving

The health managers and the health workers mentioned that they normally develop action plans after supportive supervision visits. The follow-up on the implementation of the action plans is divided into those of the health facility staff, the CHD, the implementing NGO, SMoH and sometimes the fund managers of the donor funds. Many health workers, however, noted a lack of a clear follow-up mechanism of action plans, especially where funds are required. The health workers also said that sometimes the concerned health managers either do not or delay responding to their funding requests to implement the action plan activities.

#### Context of supportive supervision

The health managers mentioned conflicts and insecurity as the major impediment to the supervision of health services in the counties. Sometimes, the supervisors must deal with both the government and armed rebel groups’ administrations to negotiate access to certain locations. Once cleared by both sides, they can go ahead with supportive supervision activities. During intercommunity violence, the supervision teams withdraw and return once the security situation normalises. The health managers also indicated other access-related challenges, such as poor road infrastructure, floods, vehicles getting stuck during the rainy season, and frequent breakdowns of the vehicles. The managers suggested that the government should improve the road infrastructures and the security and safety of citizens and humanitarian workers.

### Supportive supervision outcomes

#### Human resources management

According to the managers, the sustained discussions about staffing during supportive supervision activities have led to many counties recruiting more skilled health workers in the health facilities. A manager from Rubkona County said, *“Because of supportive supervision, we (managers) are employing people based on the qualification, unlike before when the county was a war zone”* (SSI #38). Many health managers cited some improvement in staff attendance. Some managers also observed that, although some facilities do not conform to signing attendance registers, there is considerable improvement in this aspect. To some extent, there are improved relations between the implementing NGOs, the CHD, and the SMoH because they participate in discussions and form part of the supportive supervision team. Other managers observed improved communication and cooperation among the health workers. The managers also noted the positive changes in the perceptions and attitudes of the health workers towards the patients.

#### Health information management

The managers alluded to some improvement in the completeness and timeliness of HMIS reports, data quality and availability of reporting tools in the health facilities. Through routine data audits, the quality of reporting is improving since on-the-job training is provided during supportive supervision. An SMoH official from Unity State said that *“…. although the data quality is not up-to-date, at least it has improved so much due to supervision activities”* (SSI #41).

#### Pharmaceutical management

There were mixed reactions from the participants about observed improvements in drug management. Some managers from Juba, Yei and Pariang counties mentioned that they have noticed changes in drug storage, supply, and availability as well as de-junking of expired drugs. On rational drug use, the managers mentioned that many facilities still have the challenge of prescribing drugs rationally.

#### Quality improvement

The managers cited some improvements in health service provision for some packages, such as EPI, antenatal care, and drug management in the health facilities. There is increased use of standard treatment guidelines, which to some extent has contributed to the presumed accuracy in diagnosis and drug prescription practices. There is also a noticeable improvement in some aspects of infection control and prevention, such as the segregation of medical and nonmedical waste in health facilities. For example, a manager for Torit said, *“I have seen a lot of improvement in terms of infection control and also the quality of care given to the patients but now due to the issue of payment of incentive the services are deteriorating”* (SSI #21). According to the managers, supportive supervision has helped them to look for opportunities to better manage resources (including staff) and improve the quality of care given to the patients. A CHD official from Rubkona County said that *“…. our county was among the poorest performer in terms of HMIS reporting, but now, they are catching up on reporting and on the quality of care which is attributed to supportive supervision”* (SSI #40)*.* In Wau County, it was mentioned that until recently, the health facilities that were not conducting staff meetings are now conducting them, taking minutes and filing them for the records.

## Discussion

This study has provided insights of the supervision practices in South Sudan and ascertains the contribution of supportive supervision to quality improvement in the health sector. In this section the study findings are discussed based on the notion that there are interactions between the inputs, processes, and expected outcomes of supportive supervision. Like any other system that goes through the stages of production to achieve results, supervision requires inputs/resources to facilitate the processes of production, which ultimately lead to the results. Knowledge, skills and tools are some of the key drivers that promote and improve the quality of services delivered [32]. Robust supportive supervision requires resources such as a competent health workforce with the capacity to plan, communicate, motivate, train, and build capacity within the health sector. This health workforce in turn requires financial and logistical support to execute its duties (such as money, transport, and medical equipment).

This study corroborates the observations that South Sudan is a challenging working environment and that the health sector is complicated by protracted crises, poor infrastructure, weak health workforce capacity, and inadequate health systems [4, 6, 33]. The health sector is inadequately funded and over-dependent on donors [6, 21, 34, 35]. Several contextual factors that affect the implementation of supportive supervision were identified, such as conflicts, insecurity, bad roads, and natural disasters such as floods and/or frequent breakdown of vehicles. These conditions are known to contribute to the reduction in the frequency and/or the lack of supportive supervision visits [6, 36, 37]. Due to these conditions, health workers miss out on learning opportunities and interactions with supervisors to discuss their challenges. This negatively affects the quality of health service delivery [38].

### Supervision resources/inputs

Although joint supportive supervision was conducted by a multidisciplinary team of supervisors, we found a lack of adequate and competent SMoH and CHD officials to conduct supervision. This was attributed to the high attrition of trained staff from the government departments in search of green pastures with the humanitarian organizations. This is comparable to the findings from a Pakistan study, which identified a severe lack of skills and training among the supervisors for EPI services [39]. The supervisors are expected to provide on-the-job training to health workers. Therefore, it is essential that they are well informed and trained in the general principles of supervision, including communication techniques, problem identification and solving skills as well as coaching and on-site training [15, 18, 30, 40]. This study found that most supervisors did not receive formal training in supportive supervision and health services but were instead trained on the job. Scholars argue that promoting effective supportive supervision necessitates supervisors to be knowledgeable and skilled in teaching, appraising, counselling, providing professional advice, and fostering interpersonal relationships [13, 41]. Training in supportive supervision improves supervisors’ knowledge, communication and problem-solving skills, which positively influence acceptance, motivation and job satisfaction [42–44].

Generally, health workers had positive views about the supervisors despite the delays in following up on the action plans from the relevant county authorities. However, the health workers were dissatisfied with the timing of supportive supervision sessions, especially when the supervisors go in the morning hours when the patients are many and health staff hardly have time to attend to the supervisors. The health workers acknowledged receiving coaching and mentorship on various aspects of health service delivery and quality of care, which motivates them to improve their knowledge and skills. This appears to confirm the findings from a study by De Carlo et al. [45] that suggested that supervisor honesty and responsible behaviours have a positive influence on workers’ performance and motivation.

The study findings show that supervision activities are mostly funded by donors. The funding was reported to be inadequate to support the supervision activities and to implement the action plans developed. The World Health Organization [7] proposes that to set up an effective supportive supervisory system, there is a need to ensure the availability of adequate budgets and resources. These budgets should cater for transport, fuels, allowances for supervisors and drivers, stationery, and other tools, such as checklists and smartphones/tablets, to facilitate the activities of supervisors [7, 30, 46]. The inadequate budget allocations in South Sudan limit the activities that can be implemented, including the payment of allowances for supervision. This could explain why some supervisors absent themselves from supervision visits where allowances are not provided for. A study in the Pacific region showed that the low budget allocations, coupled with the low motivation of health managers due to low salaries and limited incentives, are barriers to supportive supervision within the health systems [47]. Other studies from Gambia, Tanzania, and Zimbabwe have demonstrated that adequate budget allocations to the districts led to effective and sustainable supportive supervision practices [46, 48, 49]. Similar to the Pacific study [47], this study found that the counties lacked reliable means of transport and logistics. This means that the supervisors have limited access to vehicles (which in many cases are very old) and other logistics to enable them to undertake frequent supervision visits in their counties [46, 47]. In addition, where air travel is required, the flights are costly; therefore, frequent travel is required to supervise the locations, especially where access is challenged by insecurity and either the lack of or poor road network.

### Supervision approaches

Two main approaches were used to supervise health services in South Sudan: monitoring and supportive supervision visits. While monitoring visits were primarily focused on verifying health service delivery activities in health facilities, supportive supervision interventions were more focused on improving performance and quality of care [50, 51]. The supportive supervision interventions mostly dealt with administrative functions, and less effort was put into the supervision of clinical aspects. Embedding clinical supervision into the health system is known to be beneficial to the organisation, professional development, and patient care [52]. Supportive supervision ensures that health workers have the appropriate knowledge, skills, and motivation to deliver quality health services. Therefore, the quality of care cannot be achieved unless the healthcare providers’ clinical knowledge and skills are improved. Studies have demonstrated the benefits of supportive supervision in quality improvement. In Ethiopia and Uganda, studies have shown that supportive supervision contributes to increased adherence to standards and clinical treatment guidelines [13, 53, 54] and better management of pharmaceuticals [55]. Another study in Bangladesh found improvement in the quality of care of infants and children with sepsis due to sustained clinical supervision efforts [56]. These research findings contrast with our findings demonstrating that during supervision, little emphasis was given to improving the clinical skills of the health workers. This implies that the quality of care cannot be improved without focusing on the knowledge and skills of the health providers.

The research findings show that supportive supervision was mostly driven by the data received from health facilities, although other factors, such as emergencies, played a role in deciding the locations to supervise. Having reliable and timely data provides supervisors with the opportunity to identify areas of focus, the kind of support needed, problem-solving, and the possibility of targeted supervision [57]. The success of supportive supervision hinges on meticulous planning and coordination among the supervisors and the health workers they supervise [42]. Our findings show some gaps in the coordination among the supervisors but also with the health workers. For example, where the supervisors do not show up for supervision, where the health workers are absent, or where facilities are closed during the visits. Similar to the findings from Benin [58] and Kenya [42], the lack of coordination leads to disagreement, confusion, and wastage of scarce resources used to plan for supportive supervision visits.

The study found that supportive supervision visits were performed quarterly, while monitoring visits were monthly and/or ad hoc. There were also variations in the time spent supervising a health facility ranging from 30 minutes to 5 hours. The frequency of supervisory visits and the duration between the visits are crucial in quality improvement [50, 59]. However, there is limited evidence in the literature to show the ideal frequency and length of supportive supervision. This may be because supervision practices are not standard and cannot be compared as such, and the duration also depends on the focus and breadth of the supervision. Nonetheless, scholars report that supportive supervision should be regular [50, 52, 60]. Studies have demonstrated that supportive supervision interventions can be effective where there are regular supervisory visits to health facilities to develop relationships, monitor performance, and improve problem-solving skills [59–61]. Therefore, sufficient time investment is needed to achieve maximum benefits from supportive supervision. Studies conducted in Nigeria and Tanzania demonstrated that increasing the frequency of supervision had a positive impact on some dimensions of health service delivery, such as infrastructure, human resources for health, essential drugs and clinical practice [59, 62]. In Ethiopia, some scholars found that with regular supportive supervision, there was an improvement in pneumonia case management among under-five children [13]. However, another study from Kenya found limited evidence that increasing the frequency of supervision visits improved health service delivery [42]. Additionally, a study conducted in Northern Ghana found that regular supervision was insufficient in improving the productivity of health workers [63].

It is essential to have the right supervision tools to assist supervisors in conducting effective supervision. The study found that supervisors use various tools, but the most used was the paper-based MoH quantified supervision checklist. Other supervision tools were the Open Data Kit and the Quality of Care mobile application. Using different toolkits and/or checklists is a missed opportunity for aligning priorities in the quality of care [7, 50, 64]. Most health workers received immediate on-site verbal feedback from their supervisors, but written feedback was rare. Some health workers received neither verbal nor written feedback. Those who received feedback found it constructive and had a chance to correct their mistakes and learn new developments. These findings corroborate other studies that have highlighted the importance of feedback in promoting learning and staff development [60, 64, 65].

Our findings show that although action plans were developed at multiple levels during supportive supervision, there was a lack of clear follow-up mechanisms due to limited funds. This demotivates the health workers and deters problem-solving. For supportive supervision to be effective, a mechanism for problem-solving should be prioritised with interventions geared towards the quality of supervision, training of supervisors and development of problem-solving skills [66]. When the quality is poor, supportive supervision cannot improve the quality of services unless problems within the system are addressed [67]. Studies have shown that problem-solving supervision approaches generally lead to teamwork, motivation, skill sharing, and promote cross-learning [10, 11, 39, 40, 42].

Scholars mention performance observations, constructive feedback mechanisms, opportunities for learning and improvement, and joint problem-solving approaches as important aspects of supportive supervision [13, 18, 64]. The study findings show that the supervisors check the registers, hold discussions with the health workers, provide on-the-job training, jointly develop action plans to solve the identified problems and strengthen relationships between the supervisors and supervisees. This is comparable to what other studies found in Pakistan, where the supervisors checked registers and provided on-the-job training sessions to improve the knowledge and skills of health workers to enhance immunization services [39]. Similar to our findings, a study by Kok et al. [10] in four African countries (Ethiopia, Kenya, Malawi, Mozambique) found that supervisors check registers, and the progress of implementation of activities, discuss possible solutions to the problems identified and provide mentoring when it is needed.

### Supportive supervision outcomes

Based on the study findings, some health managers said that sustained supportive supervision efforts have resulted in the recruitment of qualified health workers, improved staff attendance and improved staff attitudes towards each other and the patients. Additionally, there is presumed improvement in health data reporting, drug management, and marginal improvement in the use of standard treatment guidelines as well as infection prevention and control measures, all attributed to the on-the-job training sessions given during supervision. Our findings corroborate those from the Mozambique study [44], where health workers perceived improvements in their performance, motivation, increased participation, and voice amongst themselves due to supportive supervision. Avortri and others [13] have also alluded to the potential of supportive supervision in improving the quality of health care and enhancing the skills of health workers. Other studies have shown that supportive supervision can increase job satisfaction and lead to the formation of relationships where trust, teamwork, and two-way communication are fostered. This consequently raises health workers’ morale, motivation and knowledge and skills towards performance and quality improvement [8, 44, 46, 51].

The insights from this study give rise to various recommendations to guide supportive supervision initiatives in South Sudan and similar settings. There is a need to enhance stewardship of the MoH and its subsidiaries at the state and county levels to take full responsibility for all supportive supervision activities. The health managers should be trained on supportive supervision approaches such as appraisal, communication, problem identification and solving skills to facilitate effective supervision of health services. Additionally, there should be timely and constructive feedback with actionable plans. Supportive supervision visits to the health facilities should be regular to enable follow-up on the issues spotted during the previous supervisory visits and implementation of the agreed action plans. The objectives of supportive supervision visits should be communicated to health workers before the visits for adequate preparations. The time dedicated to supportive supervision should be sufficient to facilitate learning and problem-solving activities. This is crucial for motivation and performance. Furthermore, to avoid duplication of efforts and align priorities, there is a need to harmonise and integrate supportive supervision tools and guides across the country. Focused supportive supervision should be encouraged based on the problems identified to ensure quality improvement. Adequate funds should be provided to facilitate the supervision processes and implementation of the action plans. Last, there should be an investment in transport (such as four wheel drive vehicles), training of the health workforce, and medical equipment to facilitate the provision of quality health services.

### Study limitations

This study was subject to several limitations. The study was susceptible to recall bias when responding to some of the FGD questions; however, the researchers tried to refer to the most recent supportive supervision visit that the participants attended. The study also did not include observations of the actual supportive supervision sessions; hence, the findings are based on the information given by the participants. Insecurity and the poor state of the road network due to floods in Kapoeta led to the cancellation of three FGDs where the researcher could not access the participants in the health facilities.

The four authors’ roles in providing technical assistance to the HPF programme and their interplay with research participants, some of whom were known to them, might have introduced certain participant biases. The participants were recruited through the SMoH and CHD networks who are also receiving some funding for health services delivery from the HPF programme, which might have led to some degree of participant bias. Some participants from the CHD, implementing NGOs, and health workers were getting either salaries or incentives from the HPF programme, and they might have assumed that giving undesirable responses could lead to a reduction in their funding. This could have resulted in being less critical about the supervision activities they are responsible for. The researchers mitigated this by reiterating that the responses they gave are treated confidentially and anonymously and will not have any impact on their employment or benefits but that instead the research was meant to gain insight into supportive supervision practices and ultimately identify areas for improvement.

## Conclusion

This study has explored the current supervision practices in South Sudan. Supportive supervision remains a daunting task in the health sector due to protracted crises, rudimentary health infrastructure, and low health workforce capacity. As presented in our findings, supervision is impacted by contextual factors such as insecurity, poor road networks, floods, inadequate funding, and poor coordination mechanisms among stakeholders. Therefore, strengthening the supervision processes and providing inputs (resources and logistics) for supervision as presented in this paper remains a priority if quality improvement is to be realised in the health sector. Enhancing supportive supervision processes has the potential to transform the quality of health care in South Sudan. However, current practices are not sufficiently harmonised and integrated into national health governance structures but are instead led and implemented by NGOs and UN agencies. To ensure sustainability, MoH stewardship, commitment, and investment in the capacity of the health workforce and health infrastructure development are required. Given the dearth of literature on supervision and health service quality in South Sudan, future studies could further explore how the knowledge and skills acquired during supportive supervision have impacted accountability, local governance, and the quality of care provided to citizens.

## Supplementary Information

**Additional file 1.** Key Informant interview guide. The quide presents the questions and probes that were used to collect data from the key informants.

**Additional file 2.** Focus group discussion guide. The focus group discussion guide contains a set of the questions and probes that were used during the focus group discussions with the health workers.

## Data Availability

All data generated or analysed during this study are included in this published article and the materials are in Supplementary file 1 – Semi-structured interview guide and Supplementary file 2 – Focus group discussion guide.

## Abbreviations

CHD: County Health Department
EPI: Expanded Programme for Immunization
FGD: Focus Group Discussion
HPF: Health Pooled Fund
HMIS: Health Management Information System
MoH: Ministry of Health
NGO: Non-Governmental Organisation
PHCC: Primary Health Care Centre
PHCU: Primary Health Care Unit
QoC: Quality of care
QSC: Quantified supervisory checklist
RERB: Research Ethics Review Board
SMoH: State Ministry of Health
SSI: Semi-structured interview
UN: United Nations
UNICEF: United Nations Children’s Fund
WHO: World Health Organization

## Footnotes

1 Boma is the lowest administrative unit of local government in South Sudan.

2 Health Pooled Fund is a multi-donor funding mechanism that consists of six donors: the United Kingdom, Canada, the European Union, Sweden, the United States, and Gavi and supports services delivery in eight of the 10 states
