# Supplementary material for: Health services supervision in a protracted crisis: a qualitative study into supportive supervision practices in South Sudan
Source: BMC Health Serv Res. 2022 Oct 14;22:1249. doi: 10.1186/s12913-022-08637-4 (PMC9568951; doi:10.1186/s12913-022-08637-4)
Supplement: Supplementary file 1 — Additional file 1. Key Informant interview guide. The quide presents the questions and probes that were used to collect data from the key informants. [file 12913_2022_8637_MOESM1_ESM.docx]

**SUPPLEMENTARY FILE 1 (S1)**

**SEMI-STRUCTURED INTERVIEW GUIDE FOR HEALTH MANAGERS**

**Introductions**

*The questions that will be answered by the managers (key informants) are outlined below. The questions are given under* ***five*** *guiding topics. Where the question is not clear please try to rephrase or explain it in a different way to the participants. The participants may respond to all questions, but in case the participant opts not to answer any of the questions, then move to the next question.*

| **Introductions and preliminary Information** | ***UID Number*** | | **Interview type_State_Code_Number_Participant type** |
| --- | --- | --- | --- |
|  | *Date of the interview and time of the interview* | |  |
|  | *Organisation of the respondent* | |  |
|  | *Gender of the respondent* | |  |
|  | *Current position* | |  |
|  | *Duration in the current position* | |  |
|  | *What is your role(s) and experience with supervision of health services? ​* | |  |
| ***In this conversation, I would like to discuss how supervision is carried out, the major challenges with supervision, and how supervision can be improved.*** | | | |
| **Topic guide, questions and probes** | | | |
| **Topic** | **Sub-topic** | **Guiding questions** | **Specific questions and probes** |
| 1. **Supervision processes** | Approach | How do you supervise the health providers? | *Probes*   - *What methods do you use, what do you focus on?* - *Who are the supervisors?* - *What is the content of supervisions (i.e., register review, data collection, observation of drugs and supplies, observation of consultations etc.)?* - *How often is supervision carried out?* - *What modes of transportation are used?* - *How much time is spent on supervising one facility/department?* - *How are the supervisors supervised? Who supervises them and how often?* |
|  | Tools | What tools do you use for supervision? | *Probes*   - *Do you use a supervision checklist?* - *How were you trained on the use of the checklist?* - *Is information recorded on paper or electronically?* - *Who do you submit information from supervision to?* - *How do you feel about the checklist? (Do you understand it well? Is it complicated? Is it time-consuming?)* |
|  | Training | What training did you receive on supervision of health services? | *Probes*   - *What topics were you trained on?* - *How long was the training?* - *Who provided the training?* |
|  | Needs assessment | How do you decide where supervision is done? | *Probes*   - *How do you decide on the areas to supervise?* - *Is there any assessment done?* - *Is it data driven?* - *Is it a reaction to a problem?* |
|  | Communication and feedback | How do you communicate the supervision findings to the supervisees? | *Probes*   - *How do you share feedback? Is verbal or written?* - *Who communicates the findings?* - *Who else do you shared the findings of your supervision? e.g. IP, SMOH, other CHD staff, etc?* - *Any tools used to give feedback?* - *Is record of supervision findings kept in the office?* - *How do the health workers perceive the feedback you give them?* |
|  | Action plans | What agreements do you make following the supervision? | *Probes*   - *Who makes the agreements or action plans?* - *Do you ever follow up to see whether these action plans are implemented?* - *How do you follow up on the action points or plans?* - *What improvements have you observed as a result of your supervision efforts?* |
| 1. **Decision-making** | Use of supervision findings | How do you use the findings from supervision? | *Probes*   - *What evidence is used?* - *Who is involved in making decisions?* - *What guidance and tools are used?* - *Are decisions explicitly recorded?* - *Are decisions reported on?* - *Are decisions ever revised?* - *What information is considered in revisions?* |
|  | Challenges with decision making | What challenges do you encounter in your decision-making processes? | *Probes*   - *How much autonomy do the health managers have on spending, HR management etc.?* - *How do you solve the problems you identify during support supervision?* |
| 1. **Challenges** |  | What challenges to do you face during support supervision? | *Probes*   - *What are the challenges to carrying out supervision? (Incentives? Time? Transport? Road infrastructure, HR? etc.)* - *What are the challenges to quality of supervision?* - *How are challenges dealt with?* |
| 1. **Opinions and Observations** |  | What changes have you observed in the counties resulting from the supervision efforts? | *Probes:*   - *staff attendance, and use of guidelines, water and sanitation, use of drugs, completeness of HMIS reports, quality of care etc****.*** |
| 1. **Suggestions for improving supervision** |  | How can supervision be improved? | - *What work well and what didn’t work well?* - *What do you want to improve your in supervision?* |
