# Supplementary material for: Health services supervision in a protracted crisis: a qualitative study into supportive supervision practices in South Sudan
Source: BMC Health Serv Res. 2022 Oct 14;22:1249. doi: 10.1186/s12913-022-08637-4 (PMC9568951; doi:10.1186/s12913-022-08637-4)
Supplement: Supplementary file 2 — Additional file 2. Focus group discussion guide. The focus group discussion guide contains a set of the questions and probes that were used during the focus group discussions with the health workers. [file 12913_2022_8637_MOESM2_ESM.docx]

**SUPPLEMENTARY FILE 2**

**FOCUS GROUP DISCUSSION (FGD) GUIDE FOR THE HEALTH FACILITY WORKERS**

**Introductions**

*The questions that will be answered by health workers are outlined below. The questions are given under* ***seven*** *guiding areas with a number of probing questions. In case the question is not clear please try to rephrase or explain it in a different way to the participants. The participants may respond to all questions, but in case some participants opt not to answer any of the questions, please respect their decision and move on to the next question (s).*

1. **Experiences with supervision**

- I would like you to think of the most recent supervision you received!
  - How was the experience with this supervision?
  - What is the content of supervision (i.e., register review, data collection, observation of drugs and supplies, observation of consultations etc.)? Did it meet your expectations? What did you appreciate? What did you not appreciate? Who were the supervisors? (From which organisations/institutions, Are they well informed? Are they like teachers, or are they like the police?)
  - Which tools did they use? - Checklists, QoC app etc?
  - How many visits were made? What was the duration of the visits?
  - How did you get to know about this supervision visit?

1. **Communication and feedback**

- How do you get to know about the findings from the supervision you get?
  - How often do you get the feedback? Is it written or verbal? Can you give examples? How is it for others?
  - What do you think about this feedback?
  - Is there a system for you to give feedback to your supervisors?

1. **Action plans and decision making?**

- Do you have any agreements on supervision and follow-up from supervisors? Give an example of some of the agreements/action point that were made from the last visit you received?
  - Are action plans developed or not? Who is involved in developing the action plans?
  - How are the action plans recorded? Are they recorded anywhere in your facility? Who keeps this record?
  - Are action plans known to everyone in the health facility/ department?
  - How do you decide on which actions plans to implement?
  - Who follows up on the action points and how is this follow up done? Are there any follow up visits?
  - What challenges face implementing these action plans?

1. **Use of supervision?**

- How does supervision help you in your work?
  - Probes: Improving skills? Improvements in staff attendance? Use of guidelines? Water and sanitation, use of drugs, completeness of HMIS reports etc?

1. **Satisfaction with supervisors?**

- How are satisfied are you with the way supervision is carried out in this county?

1. **Challenges**

- What challenges are the challenges to supervision in your facility?

1. **Suggestions for improvement**

- What are your suggestions for improving supervision in the facility/county?
  - Schedules and timing?
  - Communication and feedback?
  - Transport?
  - Skills for supervisors?
  - Decision making?
  - HR etc?

**THANK YOU FOR YOUR PARTICIPATION**
